# African Swine Fever Virus Protein pE199L Mediates Virus Entry by Enabling Membrane Fusion and Core Penetration

**DOI:** 10.1128/mBio.00789-20

**Published:** 2020-08-11

**Authors:** Tania Matamoros, Alí Alejo, Javier María Rodríguez, Bruno Hernáez, Milagros Guerra, Alberto Fraile-Ramos, Germán Andrés

**Affiliations:** a Centro de Biología Molecular Severo Ochoa, Consejo Superior de Investigaciones Científicas and Universidad Autónoma de Madrid, Madrid, Spain; b Centro de Investigación en Sanidad Animal, Instituto Nacional de Investigación y Tecnología Agraria y Alimentaria, Madrid, Spain; c Centro Nacional de Biotecnología (CNB-CSIC), Madrid, Spain; d Universidad Complutense de Madrid, Departamento de Biología Celular, Facultad de Medicina, Madrid, Spain; Centro Nacional de Biotecnologia

**Keywords:** African swine fever virus, ASFV, giant DNA virus, nucleocytoplasmic large DNA virus, NCLDV, viral fusion, virus entry, virus uncoating

## Abstract

African swine fever virus (ASFV) is a complex nucleocytoplasmic large DNA virus (NCLDV) causing a lethal hemorrhagic disease that currently threatens the global pig industry. Despite its relevance in the infectious cycle, very little is known about the internalization of ASFV in the host cell. Here, we report the characterization of ASFV protein pE199L, a cysteine-rich structural polypeptide with similarity to proteins A16, G9, and J5 of the entry fusion complex (EFC) of poxviruses. Using biochemical and immunomicroscopic approaches, we found that, like the corresponding poxviral proteins, pE199L localizes to the inner viral envelope and behaves as an integral transmembrane polypeptide with cytosolic intramolecular disulfide bonds. Using an ASFV recombinant that inducibly expresses the *E199L* gene, we found that protein pE199L is not required for virus assembly and egress or for virus-cell binding and endocytosis but is required for membrane fusion and core penetration. Interestingly, similar results have been previously reported for ASFV protein pE248R, an inner membrane virion component related to the poxviral L1 and F9 EFC proteins. Taken together, these findings indicate that ASFV entry relies on a form of fusion machinery comprising proteins pE248R and pE199L that displays some similarities to the unconventional fusion apparatus of poxviruses. Also, these results provide novel targets for the development of strategies that block the first stages of ASFV replication.

## Abstract (precis)

African swine fever virus (ASFV) causes a highly lethal swine disease that is currently present in many countries of Eastern Europe, the Russian Federation, and Southeast Asia, severely affecting the pig industry. Despite extensive research, effective vaccines or antiviral strategies are still lacking and relevant gaps in knowledge of the fundamental biology of the viral infection cycle exist. In this study, we identified pE199L, a protein of the inner viral membrane that is required for virus entry. More specifically, pE199L is necessary for the fusion event that leads to the penetration of the genome-containing core in the host cell. Our results significantly increase our knowledge of the process of internalization of African swine fever virus, which may instruct future research on antiviral strategies.

## INTRODUCTION

African swine fever virus (ASFV) causes a highly lethal hemorrhagic disease in domestic pigs and wild boars for which there is no vaccine or other therapeutic strategy available (1–3). The disease, which is endemic in sub-Saharan Africa, has spread over the last years through Eastern Europe, the Russian Federation, and Southeast Asia, seriously threatening the global pig industry (4).

ASFV is the sole known member of the family *Asfarviridae*, within the proposed order of nucleocytoplasmic large DNA viruses (NCLDV). The NCLDV superfamily is a heterogeneous though monophyletic clade of genetically and structurally complex eukaryotic viruses (5, 6) displaying broad tropism for hosts that comprise phagotrophic protists such as amoebas as well as animals such as insects, reptiles, and mammals. In addition to wild and domestic pigs, ASFV infects soft ticks of the genus *Ornithodoros*, which act as vectors and virus reservoirs. At present, ASFV is the only known DNA arbovirus.

The ASFV particle displays a unique, multilayered architecture with an overall icosahedral morphology and a diameter of about 250 nm (7–9). It comprises a genome-containing nucleoid that is successively wrapped by an icosahedral protein core shell, an inner lipoprotein membrane, an outer icosahedral protein capsid, and an outer lipoprotein membrane. The viral genome is a double-stranded DNA (dsDNA) molecule of 170 to 190 kbp containing more than 150 open reading frames (ORFs), of which about half lack any known or predictable function (10). The virion contains about 70 different polypeptides (11), including multiple components committed to virus structure as well as a set of enzymes involved in viral transcription and mRNA modification and in the maintenance of viral genome integrity.

ASFV replicates predominantly in swine monocytes and macrophages through a highly coordinated process that depends on the temporally regulated expression of different viral gene categories (12, 13). Viral DNA replication and virus particle assembly take place at specific cytoplasmic sites close to the nucleus called viral factories. Although viral morphogenesis is not completely understood, it might involve the acquisition of the innermost envelope from open single membranes derived from the endoplasmic reticulum (14). The progressive assembly of the outer capsid and the inner core shell, above and beneath the viral membrane, respectively, results in enclosure of the genome-containing material to give rise to the intracellular icosahedral mature virus (15). These particles then move toward the plasma membrane, where they acquire their outer envelope by budding from actin-dependent membrane protrusions (16).

Little is known about the molecular mechanisms underlying ASFV entry. In general, enveloped viruses use membrane fusion to gain access to the interior of the host cells (17–20). Following their binding to specific cell surface receptors, some enveloped viruses are able to fuse directly their viral envelope with the plasma membrane. Most frequently, virus particles are first internalized by endocytosis to later fuse with the endosomal membrane. ASFV uptake in pig macrophages takes place by either macropinocytosis or clathrin-mediated endocytosis (21–23) through unknown cellular receptors. Once internalized, the incoming particles move along the endolysosomal pathway (23, 24) while undergoing profound structural changes that are part of their uncoating program (23). Thus, the endocytosed virions are partially disrupted at late endosomes, losing their outer membrane and its outer capsid. This disassembly process is driven by the acidic endosomal pH and results in the exposure of the inner envelope, which then merges with the limiting endosomal membrane to release the genome-containing core into the cytoplasm (23). This internalization pathway therefore implies that proteins located at the inner viral membrane are required for fusion, while proteins located at the outer envelope might be involved in virus attachment and endocytosis.

Most enveloped viruses employ one or two membrane glycoproteins to accomplish the various stages of virus entry (e.g., influenza virus hemagglutinin [HA], vesicular stomatitis virus [VSV] G protein, or human immunodeficiency virus [HIV] env protein) (18, 20). However, some large DNA viruses employ multiple transmembrane proteins with different specific roles in the entry steps. Thus, herpesvirus internalization depends essentially on the heterodimeric gHgL complex whereas membrane fusion is mediated by the gB glycoprotein (25).

An extreme example of complexity is represented by vaccinia virus (VACV), the best-studied member of the NCLDV family *Poxviridae*, which requires at least 4 viral proteins for cell binding and another 11 viral polypeptides for the subsequent fusion event (26–28). Thus, the nonglycosylated transmembrane VACV proteins A16, A21, A28, F9, G3, G9, H2, J5, L1, L5, and O3, all of which are localized in the membrane that encloses the viral core, form an entry fusion complex (EFC) that is required for membrane fusion and core penetration. The EFC components are essential for VACV replication, albeit not for virus morphogenesis, and can be coimmunoprecipitated from infected cell membranes, indicating that they are organized in higher-order structures (26, 28). Interestingly, some poxviral EFC components show weak but significant sequence similarity and overall domain organization with putative transmembrane proteins encoded by viruses from other NCLDV families, including *Asfarviridae* (5, 29, 30). Thus, VACV paralogous proteins L1 and F9, which are also structurally related (31, 32), resemble to some extent ASFV protein pE248R. Currently, pE248R is the only inner envelope component that has proven to be essential for membrane fusion and cytoplasmic core delivery but not for ASFV assembly (23, 33). The only other VACV EFC members conserved among the different NCLDV families are three cysteine-rich transmembrane polypeptides, A16, G9, and J5, which form intramolecular disulfide bonds within the cytoplasm (28). Remarkably, a late-expressed structural ASFV polypeptide, pE199L (34), shows weak sequence identity with these VACV paralogues (5, 23), raising the possibility that it may be involved in ASFV entry.

In the present study, we addressed the role of ASFV protein pE199L in virus replication. Our results show that pE199L is an inner envelope transmembrane polypeptide containing intramolecular disulfide bonds that is required for viral core entry but not for virus morphogenesis. The presence in the ASFV particle of a second orthologue of the poxviral fusion machinery strongly suggests that the members of both virus families, and perhaps other enveloped NCLDVs, use similar strategies for core penetration. Also, our study identified a new viral target for the development of anti-ASFV strategies that block the first stage of the infectious cycle.

## RESULTS

### ASFV protein pE199L is a type I transmembrane protein of the inner viral envelope with cytoplasmic intramolecular disulfide bonds.

**FIG 1:**
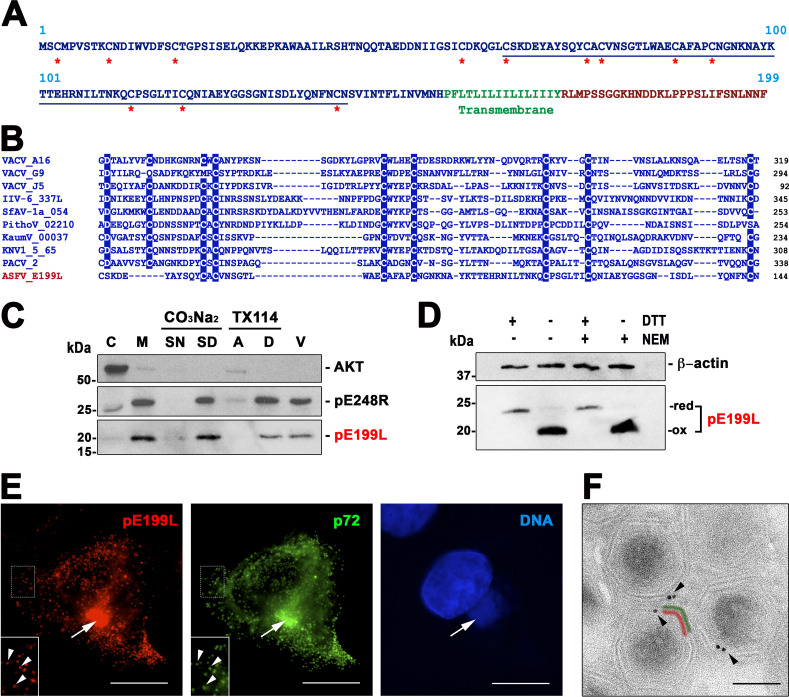
ASFV transmembrane protein pE199L. (A) Amino acid sequence of pE199L protein (BA71V strain). The putative cytosolic, cystein-rich (asterisks) N-terminal region is indicated in blue, whereas the putative transmembrane domain and the short C-terminal region are indicated in green and red, respectively. (B) Multiple-sequence alignment of the most highly conserved region (underlined in panel A) of the N-terminal region of pE199L with possible NCLDV orthologues identified in different virus families or groups as follows: poxviruses (VACV ORFs A16, G9, and J5), iridoviruses (IIV type 6, ORF IIV6-337L), ascoviruses (SfAV-1a, ORF 054), pithoviruses (PithoV ORF 02210), kaumoebaviruses (KaumV ORF 00037), mimiviruses (KNV1 type 5, ORF 65), and pacmanviruses (PACV, ORF 2). Amino acids identical in at least 80% of the selected 10 aligned sequences are highlighted. (C) Membrane association of pE199L protein. ASFV-infected cells were fractionated into cytosolic (C) and membrane/particulate (M) fractions, which were analyzed by Western immunoblotting with an anti-pE199L antibody. In addition, the membrane fraction was subjected to alkaline carbonate (pH 11.5) extraction and centrifugation or to temperature-induced phase separation after Triton X-114 extraction. Equivalent fractions of the supernatant (SN) and sediment (SD) obtained after carbonate extraction and of the aqueous (A) and detergent-rich (D) phases obtained after TX-114 extraction were analyzed as described above. Cytosolic marker AKT and membrane protein pE248R were also analyzed as controls. Purified ASFV particles (V) were also analyzed as indicated at the right of the immunoblots. Molecular masses (in kilodaltons) are indicated at the left of the blots, while the position of the pE199L band is indicated at the right. (D) pE199L forms intramolecular disulfide bonds. ASFV-infected Vero cell extracts were lysed under reducing (DTT) and nonreducing conditions in the presence or absence of the alkylating agent NEM. Protein pE199L was detected by Western blotting as described above. The positions of the bands corresponding to the oxidized (ox) and reduced (red) forms of pE199L are indicated on the right. Actin b was analyzed as a loading control. (E) Subcellular localization of pE199L. ASFV-infected cells were fixed at 18 hpi and immunolabeled with a rat antibody against pE199L (red) and MCP p72 (green). Nuclear and viral DNA was stained with Hoechst 33258 (blue). The arrows indicate colocalization at virus factories, whereas the arrowheads (insets) indicate colocalization at virus particles scattered throughout the cytoplasm. Bars, 5 μm. (F) Subviral localization of pE199L. ASFV-infected cells were fixed at 18 hpi and immunolabeled with a rat antibody against pE199L followed by an anti-rat antibody conjugated to 10-nm-diameter gold particles. Note that most of the gold particles present on intracellular viruses were associated with the inner envelope (red) beneath the outer protein capsid (green). Bar, 100 nm.

ASFV protein pE199L, also called j18L, was previously characterized as a 20-kDa virion protein that is expressed at late times after infection and localized in the virus assembly sites (34). Its amino acid sequence, which is highly conserved among different ASFV strains, contains a long cysteine-rich N-terminal segment, a potential transmembrane domain, and a short C-terminal tail (Fig. 1A). The E199L ORF was initially included in a cluster of orthologous genes identified in NCLDVs (5) which encompassed the genes encoding type I transmembrane VACV EFC proteins A16, G9, and J5 (Fig. 1B). Upon sequence alignment, total percent identity of pE199L compared to these three paralogues was found to range from 17.2% to 21.3%, within the range of identity values found among the VACV proteins (18.5% to 24.6%). Additional members of this cluster were found in NCLDV families *Mimiviridae*, *Ascoviridae*, and *Iridoviridae* (35), and blastp searches have identified further potential orthologues in the more recently described *Pithovirus*, *Pacmanvirus*, and *Kaumoebavirus* genera (Fig. 1B), which belong to the “extended *Asfarviridae*” group of NCLDVs (6). Interestingly, an alignment of representative sequences (Fig. 1B) showed that the sequence similarities are mainly limited to several conserved cysteine residues that, in the case of the VACV paralogues, were previously shown to be involved in the formation of intramolecular disulfide bonds within the cytoplasm by a virally encoded redox system (36, 37).

To analyze the biological role of protein pE199L, we first studied its interaction with lipid membranes. For this purpose, ASFV-infected cells were subjected to subcellular fractionation at 20 h postinfection (hpi) and the cytoplasmic membrane and cytosolic fractions were analyzed by Western blotting with a rat anti-pE199L serum. As a control, the cytosolic cellular kinase Akt and the viral transmembrane protein pE248R were also analyzed. As shown in Fig. 1C, protein pE199L was detected mainly in the membrane fraction. When this fraction was subjected to alkaline carbonate extraction, a treatment that dissociates peripheral proteins from lipid membranes, protein pE199L remained associated with the membrane sediment (Fig. 1C). Moreover, when the membrane fraction was subjected to Triton X-114 extraction and temperature-induced phase separation, protein pE199L partitioned entirely into the detergent phase. Taken together, these results indicate that protein pE199L behaves as an integral membrane protein, in agreement with the presence of a C-terminal hydrophobic region (Fig. 1A).

To ascertain whether protein pE199L forms intramolecular or intermolecular disulfide bonds, ASFV-infected cells were lysed under reducing and nonreducing conditions and analyzed by Western blotting using an anti-pE199L antibody. As shown in Fig. 1D, a slight but significant reduction of the mobility of the reduced pE199L protein (apparent mass, ∼23 kDa) was observed compared with the unreduced form (∼20 kDa). The same mobility shift was found when the infected cells were lysed in the presence of N-ethylmaleimide (NEM), a sulfhydryl alkylating reagent used to prevent aberrant disulfide bond formation after denaturation (Fig. 1D). No extra bands suggesting disulfide-bonded oligomers were observed. These results indicate that the majority of the pE199L protein exists as an oxidized form involving intramolecular disulfide bonds, as previously found for its VACV orthologues. We then explored the subcellular localization of pE199L in ASFV-infected Vero cells by immunofluorescence and immunoelectron microscopy. As shown in Fig. 1E, fluorescence of pE199L was detected mainly within the perinuclear viral factories, as previously reported (34), but also as punctate structures scattered throughout the cytoplasm. This pattern most likely corresponds to single virus particles, as suggested by its colocalization with the major capsid protein (MCP) p72 (ORF B646L) (Fig. 1E). To determine the subviral localization of pE199L, immunoelectron microscopy was performed on ultrathin thawed cryosections of ASFV-infected Vero cells fixed at 18 hpi. As shown in Fig. 1F, specific pE199L labeling was clearly associated with the periphery of intracellular mature particles at the viral factories. The quantification of the signal indicated a radial distribution of the gold particles of 87 nm ± 24 nm (*n* = 50), which fits well with the position of the inner envelope (95 ± 5 nm), the only lipid membrane of the intracellular virus particle.

### ASFV protein pE199L is essential for virus replication.

**FIG 2:**
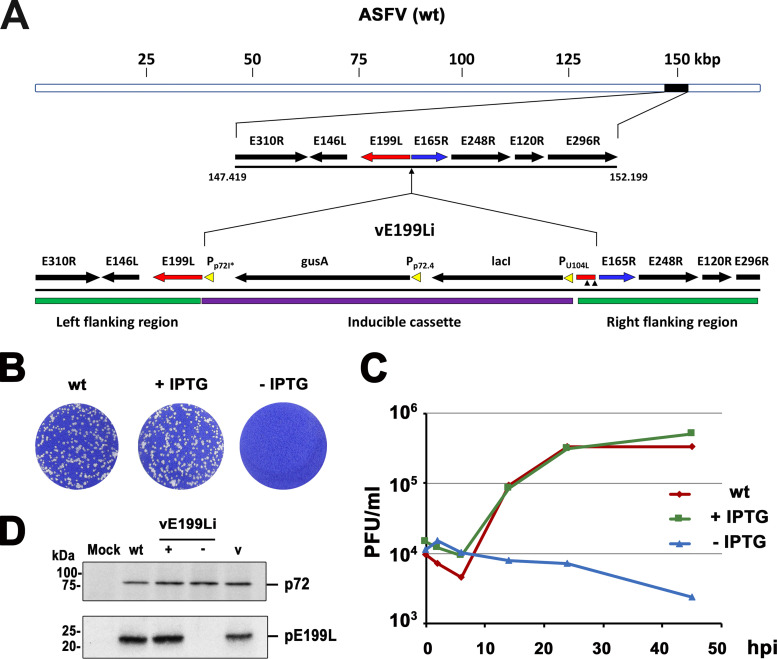
Conditional lethal phenotype of an ASFV recombinant with an inducible *E199L* gene copy. (A) Genomic structure of recombinant vE199Li. The inducible virus was obtained by homologous recombination of the parental ASFV genome (wt) with an inducible cassette containing a late, IPTG-dependent strong promoter (p72I*) for the *E199L* gene; a copy of the E. coli *lac* repressor gene (lacI); and a reporter gene (*gusA*) used for selection and purification of the recombinant virus. ASFV genes present at the left and right flanking regions are indicated. Two small vertical arrowheads indicate two point mutations introduced to remove start codons in the short sequence of E199L that had been duplicated in the right flanking region. (B) Plaque phenotype of vE199Li. Vero cell monolayers were infected with parental BA71V (wt) or recombinant vE199Li viruses in the presence (+) or absence (−) of 1 mM IPTG. Lysis plaques were visualized by crystal violet staining 5 days after infection. (C) One-step growth curves of vE199Li virus. Vero cells were infected with 5 PFU of vE199Li virus per cell in the presence or absence of IPTG. At the indicated times of infection, the virus titer of each sample was determined by plaque assay on Vero cells in the presence of the inducer. Parental BA71V (wt) infections were also titrated as a control. (D) Inducible expression of protein pE199L. Vero cells were either mock infected (mock) or infected with parental BA71V (wt) or recombinant vE199Li viruses in the presence (+) or absence (−) of IPTG. At 18 hpi, the cells were lysed and analyzed, along with purified ASFV particles (v), by immunoblotting performed with antibodies against viral proteins pE199L and p72 (loading control). The positions of the detected proteins are indicated on the right. Molecular masses are indicated on the left (in kilodaltons).

To investigate the role of protein pE199L in viral growth, we generated an inducible recombinant virus (vE199Li) in which the expression of *E199L* gene is under the control of the Escherichia coli *lac* operator/repressor system (Fig. 2A). For this purpose, the ASFV genome (BA71V strain) was modified by replacing the original *E199L* gene promoter by a late, IPTG (isopropyl-β-d-thiogalactopyranoside)-dependent promoter and by inserting the E. coli *lac* I repressor gene under the control of a constitutive promoter (38).

As shown in Fig. 2B, in the presence of 1 mM IPTG, the sizes and numbers of lysis plaques were similar for the parental and recombinant viruses, while omission of the inducer caused a drastic decrease in plaque formation by the recombinant virus. In a further approach, one-step growth curve analyses of vE199Li were performed in the presence or in the absence of 1 mM IPTG. As shown in Fig. 2C, the vE199Li growth curve obtained under permissive conditions was similar to that obtained with parental BA71V virus. In contrast, under nonpermissive conditions, the vE199Li titers were reduced by more than 2.0 log units at 48 hpi (Fig. 2C). Taken together, the results from the plaque assays and one-step growth curve analyses indicate that recombinant vE199Li is an IPTG-dependent lethal conditional mutant.

To test the inducible expression of protein pE199L, we analyzed by Western blotting the lysates of Vero cells infected with parental BA71V or recombinant vE199Li in the absence or presence of 1 mM IPTG. As shown in Fig. 2D, the expression levels of pE199L were similar in the infections with the parental virus and the recombinant vE199Li virus grown in the presence of the inducer. In contrast, pE199L was virtually undetectable when the recombinant was grown in the absence of IPTG. As a control, the levels of expression of MCP p72 were similar under all infection conditions. In summary, these results show that the conditional lethal phenotype of recombinant vE199Li correlates with the inducer-dependent expression of protein pE199L.

### Protein pE199L is not required for virus morphogenesis and egress.

**FIG 3:**
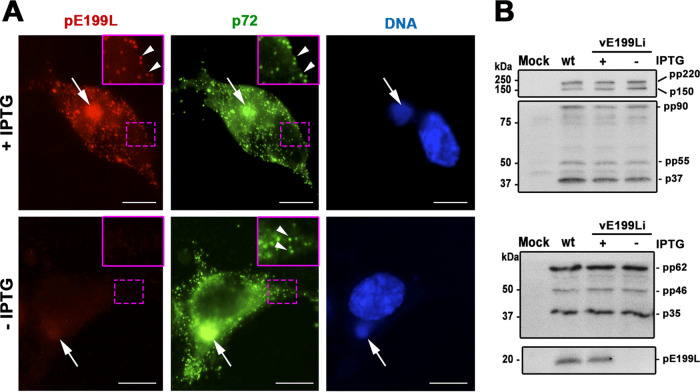
Expression of pE199L is not required for MCP p72 assembly or for proteolytic processing of core polyproteins. (A) Immunofluorescence labeling of protein pE199L (red) and MCP p72 (green) in vE199Li-infected cells at 18 hpi in the presence or absence of IPTG. Nuclear and viral DNA was stained with Hoechst 33258 (blue). The arrows indicate virus factories, whereas the arrowheads (insets) indicate virus particles scattered through the cytoplasm. Bars, 5 μm. (B) Vero cells were mock infected (Mock) or infected with parental BA71V (wt) or recombinant vE199Li viruses in the presence (+) or absence (−) of 1 mM IPTG. At 18 hpi, the cells were lysed and analyzed by immunoblotting with antibodies against the viral mature proteins p150 and p37, which were derived by proteolytic processing from polyprotein pp220, and p35, which was derived from processing of the pp62 precursor. The intermediate processing products pp90, pp55, and pp46 are also indicated on the right. Molecular masses are indicated on the left (in kilodaltons).

To study the role of pE199L protein in viral assembly, we first investigated the subcellular distribution of pE199L and the MCP p72 in permissive and nonpermissive vE199Li infections (Fig. 3A). In the presence of the inducer, both anti-pE199L and anti-p72 antibodies strongly labeled the perinuclear assembly sites as well as individual virus particles scattered throughout the cytoplasm. When pE199L synthesis was abrogated, the p72 pattern was identical to the one observed under conditions of permissive infections, indicating that protein pE199L is not required for the incorporation of MCP p72 into virus particles.

We next studied whether the lack of expression of pE199L protein interferes with virus maturation by a biochemical approach. It is known that the proteolytic processing of two viral core polyproteins, pp220 (ORF CP2475L) and pp62 (ORF CP530R), is a sensitive biochemical indicator of proper virus maturation. Indeed, defective virus assembly is often paralleled by impaired proteolytic processing, as reported for a number of inducible recombinant viruses for different virion proteins (39–43). We therefore analyzed whether polyprotein processing was affected in infections with vE199Li virus under restrictive conditions. As shown in Fig. 3B, in the absence of the inducer, the extent of polyprotein processing of pp220 and pp62 precursors was similar to that observed in the presence of IPTG or in control BA71V-infected cells. This result indicates that the lack of pE199L expression did not impair the proteolytic maturation of the core shell precursors.

**FIG 4:**
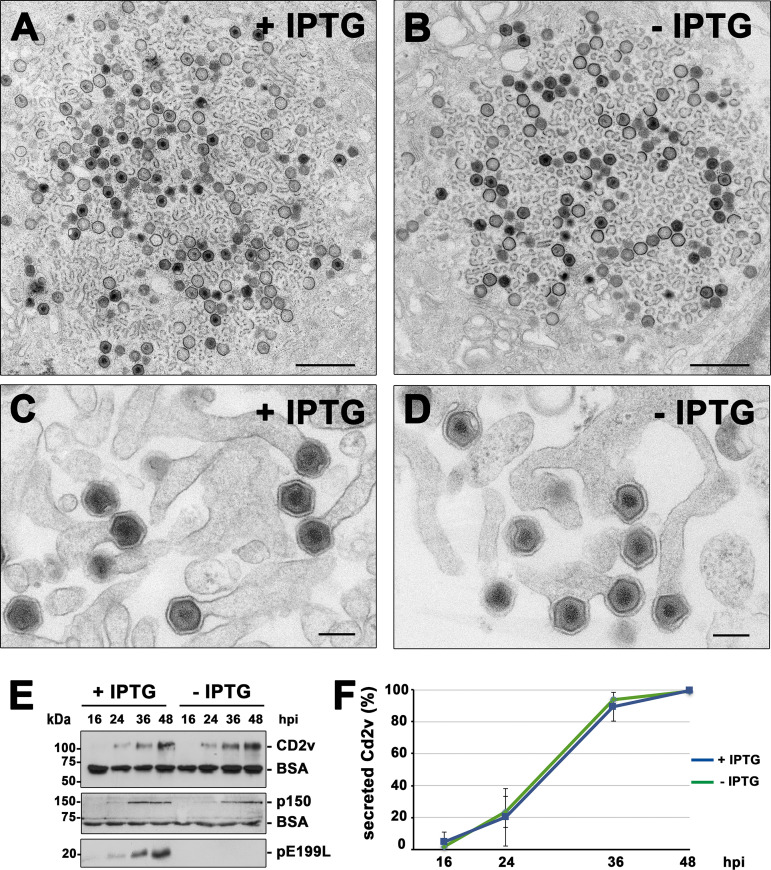
Protein pE199L is not required for virus assembly and egress. (A to D) Transmission EM of ultrathin sections of vE199Li-infected Vero cells incubated for 18 h with (A and C) or without (B and D) IPTG. Note that the overall appearance of the cytoplasmic viral factories (A and B), with the presence of numerous precursor viral membranes as well as immature and mature icosahedral intracellular particles, was similar under both sets of conditions. As shown in panel D, defective vE199Li^−^ particles apparently undergo normal budding at the plasma membrane, as occurs with the mature particles produced under permissive conditions (C). Bars, 1,000 nm (A and B) and 200 nm (C and D). (E) Supernatants from Vero cells infected with recombinant vE199Li in the presence (+) or absence (−) of IPTG were collected at the indicated times of infection and analyzed by immunoblotting for the outer viral envelope protein CD2v, the core shell protein p150, and the inner envelope protein pE199L. An antibody against BSA was used as a loading control. (F) Quantification by densitometry of the CD2v bands detected by immunoblotting as described for panel E. Data are expressed as percentages of secreted CD2v (means ± SD of results from triplicate samples) relative to that reached at 48 hpi in a vE199Li infection in the presence of IPTG. Data were normalized according to the levels of BSA detected under each set of conditions.

We then carried out an ultrastructural analysis of vE199Li-infected Vero cells at late times of infection (18 hpi) by transmission electron microscopy (EM). As shown in Fig. 4A and B, the cytoplasmic viral factories looked similar under permissive and restrictive conditions, displaying similar proportions of viral membrane precursors, icosahedral virus intermediates, and complete intracellular particles. Importantly, no ultrastructural differences were detected in comparisons of the mature vE199Li particles produced under permissive and restrictive conditions. Moreover, the budding events of the recombinant virus at the plasma membrane appeared similar under the two sets of conditions (Fig. 4C and D).

To investigate whether repression of pE199L synthesis affects virus egress, we analyzed the expression levels of two virion polypeptides, the outer membrane protein CD2v and the core shell protein p150, in the infection supernatants at different time points. Western blotting showed that those two proteins were released into the extracellular space with similar kinetics and at similar levels under both permissive and restrictive conditions (Fig. 4E and F). Taken together, these results indicate that protein pE199L is not required for virus assembly and exit.

### Protein pE199L is required for an early step of ASFV infection.

**FIG 5:**
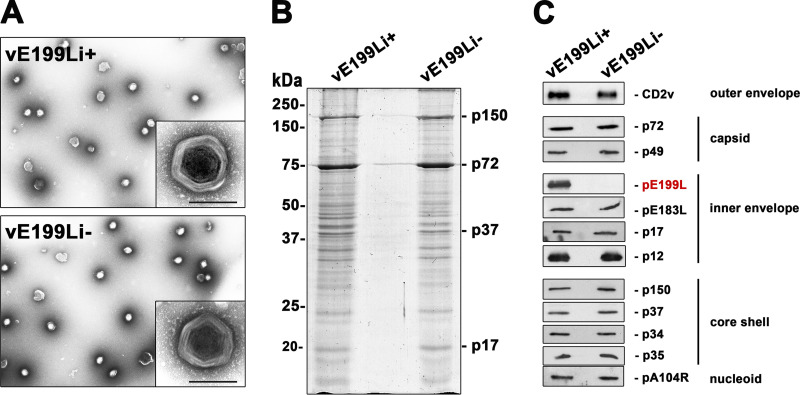
Purification and protein composition of viral particles lacking pE199L. Extracellular vE199Li virus particles generated in the presence or absence of 1 mM IPTG were obtained from infection supernatants and purified by the use of Percoll density gradients. (A) EM micrographs of negatively stained control vE199Li^+^ and defective vE199Li^−^ purified virus particles. Insets show details at a higher magnification. Bars, 200 nm (insets). (B) Equal amounts of control vE199Li^+^ and defective vE199Li^−^ particles were subjected to SDS-polyacrylamide gel electrophoresis analysis and Coomassie blue staining. Note that the samples display similar overall protein patterns. (C) Equal amounts of vE199Li^+^ and vE199Li^−^ virions were analyzed by immunoblotting with a panel of antibodies against some representative major ASFV structural proteins located at the five virus structural domains (indicated on the right). No differences were detected except for the absence of pE199L protein (in red) in vE199Li^−^ particles.

Since protein pE199L is essential for ASFV viral growth but not for virus morphogenesis and egress, we studied the infectious capability of viral particles lacking pE199L. For this purpose, extracellular vE199Li viral particles produced under nonpermissive conditions (termed “defective vE199Li^−^ particles” throughout this paper) were purified by Percoll density gradients from infection supernatants. As a control, the extracellular particles produced under permissive conditions (termed “control vE199Li^+^ particles”) were also analyzed. The viral particles containing pE199L and those lacking pE199L banded at the same gradient position and looked similar in negative-staining EM analysis (Fig. 5A). Moreover, the two virus preparations displayed nearly identical electrophoretic profiles when separated on SDS-polyacrylamide gels followed by Coomassie blue staining (Fig. 5B). In addition, Western blotting analyses showed that the control vE199Li^+^ and defective vE199Li^−^ particles contained similar levels of several major virion proteins located at the five structural virus layers, with the exception of protein pE199L, which was absent in defective vE199Li^−^ particles (Fig. 5C).

**FIG 6:**
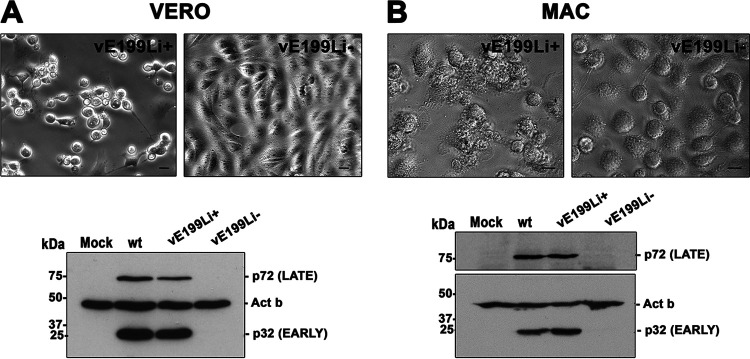
Viral particles lacking pE199L do not replicate in Vero cells or in swine macrophages. Vero cells (VERO) or swine macrophages (MAC) were infected for 24 or 16 h, respectively, with equal amounts of control vE199Li^+^ (5 PFU/cell) or defective vE199Li^−^ virus particles. Infections were performed in the presence of IPTG. (Upper panels) Phase-contrast imaging shows a clear cytopathic effect in vE199Li^+^ infections but not in vE199Li^−^ infections in Vero cells (A) and macrophages (B). Bars, 5 μm. (Lower panels) Western immunoblotting of extracts of Vero cells (A) and swine macrophages (B) infected as described above was performed using antibodies against early p32 and late p72 viral proteins. Mock-infected cells and cells infected with the parental virus (wt) were also analyzed. The expression level of β-actin (Act b) was used as a loading control. Molecular masses are indicated on the left (in kilodaltons).

Next, we infected Vero cells and porcine macrophages with equivalent amounts of purified vE199Li^+^ and vE199Li^−^ particles and studied the occurrence of cytopathic effect by phase-contrast microscopy and the expression levels of early and late viral proteins by Western blotting. As shown in the upper panels in Fig. 6, whereas control vE199Li^+^ particles induced extensive morphological changes in both Vero cells and swine macrophages, no signs of cytopathic effect (including cell detaching and cell rounding) were seen in the cells infected by defective vE199Li^−^ particles. Moreover, the cells infected by defective vE199Li^−^ particles did not express either early (p32/pCP204L) or late (p72) viral markers, which were detected in infections with control vE199Li^+^ and parental BA71V viruses (Fig. 6, bottom panels). Collectively, these results indicate that the infection by the virus particles lacking protein pE199L was arrested prior to early viral gene expression.

### Protein pE199L is not required for cell attachment and endocytosis.

**FIG 7:**
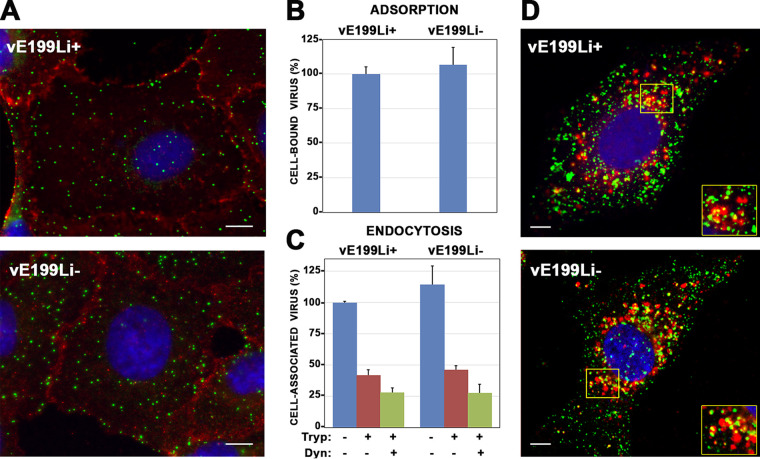
Protein pE199L is not required for virus binding and endocytosis. (A) Vero cells were incubated with equivalent amounts (5 PFU/cell) of control vE199Li^+^ and defective vE199Li^−^ virus particles during 2 h at 4°C. After extensive washing, the cells were fixed with methanol and analyzed by immunofluorescence for MCP p72 (green) and cellular plasma membrane protein p24 (red). Nuclei were stained with Hoechst 33258 (blue). Bars, 5 μm. (B) Quantitation of virus adsorption of control vE199Li^+^ and defective vE199Li^−^ particles by immunofluorescence. The number of bound virus particles per cell under each set of conditions (typically 50 to 150 virus/cell and 30 cells per condition) was counted using maximum intensity projections from the immunofluorescence experiment described above. Data are expressed as percentages of virus adsorption relative to control vE199Li^+^ infections (means of results from three independent experiments ± SD). (C) Quantitation of virus uptake of control vE199Li^+^ and defective vE199Li^−^ particles by flow cytometry. Vero cells were incubated with equal amounts (10 PFU/cell) of control vE199Li^+^ and defective vE199Li^−^ virus particles during 2 h at 37°C, in the presence or absence of 50 mM Dynasore (Dyn), an endocytosis inhibitor. Then, the cells were treated, or not, with trypsin (Tryp) to remove noninternalized particles and immunolabeled with an antibody against ASFV protein p12 followed by a fluorescent secondary antibody. Fluorescence was quantified by flow cytometry, and data are displayed as mean values (relative to control vE199Li^+^ infections in the absence of Dynasore and trypsin) and SD of results from triplicate samples. (D) Endocytosed defective vE199Li^−^ particles localize in CD63^+^ late endosomes and lysosomes. Vero cells were infected with control vE199Li^+^ and defective vE199Li^−^ viruses for 2 h at 37°C. After fixation with PFA, cells were stained with antibodies against inner membrane protein p12 (green) and the late endosomal and lysosomal marker CD63 (red). Insets show colocalization areas for both conditions. Bars, 5 μm.

To determine the stage of the replicative viral cycle that is blocked during the infection by defective vE199Li^−^ virus particles, we analyzed their ability to bind to and to be endocytosed by host cells. To address the first issue, equivalent amounts of control vE199Li^+^ and defective vE199Li^−^ particles were incubated with Vero cells for 2 h at 4°C. After extensive washing, the cells were fixed and the viruses bound to the cell surface were analyzed by immunofluorescence with antibodies against MCP p72. To better identify the outline of the infected cells, an antibody against the plasma membrane cellular marker p24 was employed (Fig. 7A). Quantitative analysis showed similar levels of membrane-bound particles under both sets of infection conditions (Fig. 7B).

To analyze uptake of the virus, equivalent amounts of control vE199Li^+^ and defective vE199Li^−^ particles were incubated with Vero cells for 2 h at 37°C to allow internalization followed by a trypsin treatment to remove any remaining surface-bound virus particles. Fixed cells were stained with an antibody against the viral inner envelope protein p12 (ORF O61R) (11) followed by a fluorescent secondary antibody. Finally, the virus-associated fluorescent signal was quantified by flow cytometry using nontrypsinized infected cells as a reference for the total amount of cell-associated particles. As shown in Fig. 7C, the amounts of internalized viruses were similar for the control vE199Li^+^ particles and defective vE199Li^−^ particles. Moreover, similar reductions in the uptake of both virus particles were observed when the experiment was performed in the presence of 50 mM Dynasore (Fig. 7C), a dynamin inhibitor that partly inhibits ASFV entry in Vero cells (23, 44). Finally, we analyzed whether endocytosed defective vE199Li^−^ particles are able to reach late endosomes, the cellular compartment where the incoming particles undergo membrane fusion (23). With this aim, Vero cells were incubated with equivalent amounts of either control vE199Li^+^ or defective vE199Li^−^ particles for 2 h at 37°C. Then, fixed cells were subjected to double staining with antibodies against the inner envelope viral protein p12 and the late endosomal and lysosomal cellular marker CD63. Immunofluorescence microscopy showed strong and comparable levels of colocalization of p12^+^ virus particles with CD63^+^ endosomes in the control vE199Li^+^ and defective vE199Li^−^ infections (Fig. 7D). A quantitative analysis of the p12^+^ and CD63^+^ signals showed similar Manders colocalization coefficients (M) for the two conditions (for the VE199Li^+^ particles, M[p12] = 0.62 ± 0.09 and M[CD63] = 0.36 ± 0.11; for the vE199Li^−^ particles, M[p12] = 0.65 ± 0.08 and M[CD63] = 0.28 ± 0.12). Taken together, these results indicate that protein pE199L is not necessary for proper attachment and internalization of ASFV.

### Protein pE199L is required for membrane fusion and core penetration in the cytoplasm.

**FIG 8:**
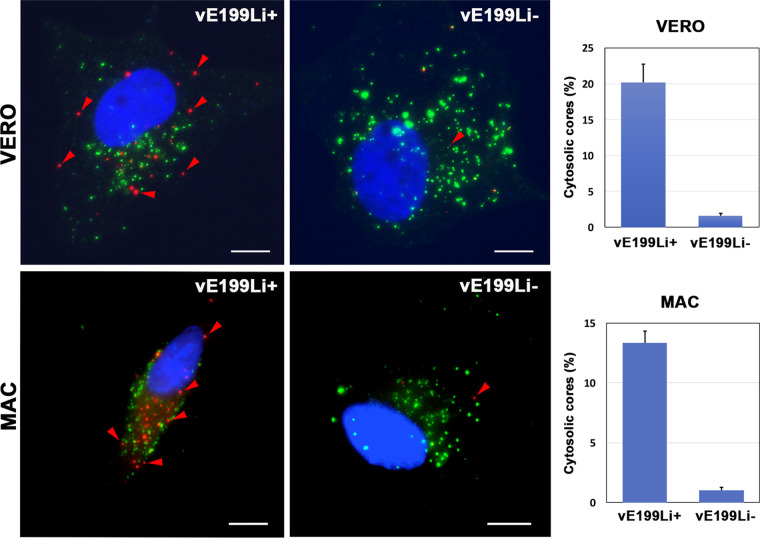
Protein pE199L is required for core penetration. Vero cells (upper panels) or swine macrophages (bottom panels) were infected with equivalent amounts (25 PFU/cell) of control vE199Li^+^ and defective vE199Li^−^ virus particles during 3 h at 37°C. After fixation, the cells were permeabilized with saponin and immunostained with antibodies to inner envelope protein p12 (green) and core shell protein p150 (red). Nuclei were stained with Hoechst 33258 (blue). Bars, 5 μm. Note that levels or red punctate p12^−^ p150^+^ structures, which are interpreted as cytosolic viral cores, are drastically reduced in vE199Li^−^ infections. The proportion of cytosolic cores for each condition and cell type is expressed as a percentage (means ± SD of results from triplicate samples) of the total particles detected per cell (right panels).

As mentioned before, endocytosed ASFV particles fuse their inner envelope with the limiting membrane of late endosomes, which results in the delivery of nonenveloped genome-containing cores to the cytosol (23). To ascertain if protein pE199L plays a role in core penetration, we quantified the appearance of “naked” cores in the cytoplasm of control vE199Li^+^ and defective vE199Li^−^ infections by immunofluorescence microscopy. With this aim, infected Vero cells or swine macrophages were fixed at 3 hpi (a time point when maximal accumulation of cytosolic viral cores is observed [23]) and then permeabilized with 0.1% saponin. Then, the cells were subjected to double staining with antibodies against the viral inner membrane protein p12 and the viral core component p150 derived from polyprotein pp220 (45). In this assay, the occurrence of p12^−^ p150^+^ punctate structures was interpreted as indicative of core penetration whereas the presence of either p12^+^ p150^−^ or p12^+^ p150^+^ particles was interpreted as indicative of the presence of cell-bound extracellular viruses along with endocytosed particles (Fig. 8). Importantly, while p12^−^ p150^+^ particles were abundantly detected in infections with control vE199Li^+^ particles, their presence in infections with defective vE199Li^−^ particles was drastically reduced. Indeed, a quantitative analysis showed that under the latter conditions, the proportion of presumptive naked cores was more than 12 times lower in both Vero cells and swine macrophages (Fig. 8, right panels). These results strongly indicate that core delivery to the cytoplasm was severely impaired in the infection by defective vE199Li^−^ viral particles.

**FIG 9:**
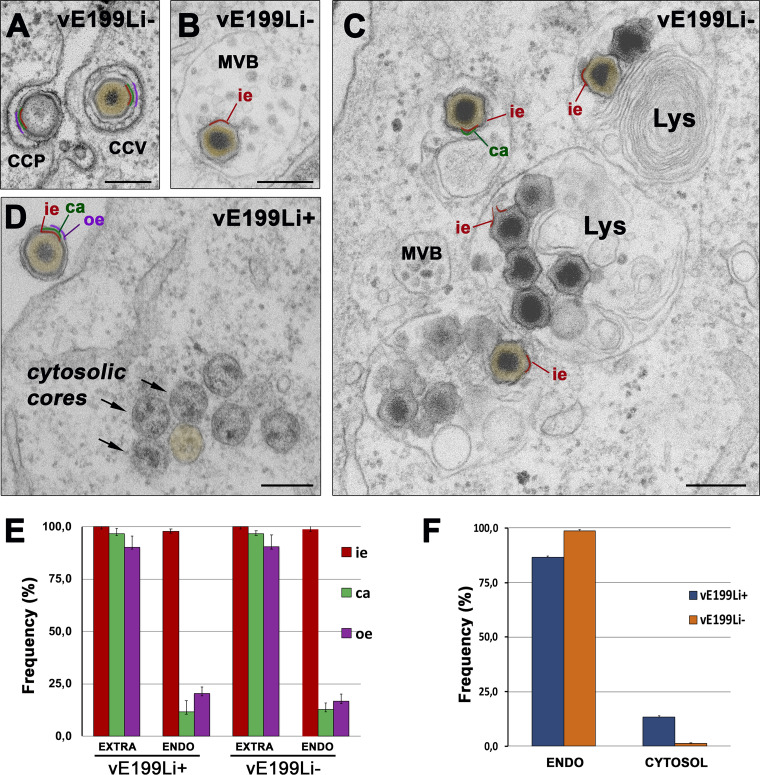
Protein pE199L is necessary for viral membrane fusion. (A to D) Vero cells were infected with equivalent amounts (100 PFU/cell) of control vE199Li^+^ and defective vE199Li^−^ purified virus particles. At 3 hpi, the infected cells were fixed, embedded in resin, and examined by EM. Note that defective vE199Li^−^ particles undergo normal endocytosis through clathrin-coated pits (CCP) and clathrin-coated vesicles (CCV) (A) and transit to late multivesicular bodies (MVB) (B) to accumulate eventually in lysosome-like structures (Lys) (C). Results of endosomal virus disassembly, which involves the loss of the outer envelope (oe, depicted in purple) and the outer capsid (ca, green), were similar for control vE199Li^+^ and defective vE199Li^−^ particles. However, the delivery of viral cores (brown) into the cytosol after inner envelope (ie; red) fusion was severely impaired for defective vE199Li^−^ particles, in contrast to that observed for vE199Li^+^ particles (D). Bars, 200 nm. (E) Quantification of virus disassembly. The endocytosed (ENDO) control vE199Li^+^ and defective vE199Li^−^ particles (*n* > 100 per condition) were classified according to their layer content (ie, inner envelope; ca, capsid; oe, outer envelope). As a reference, the layer content of membrane-bound extracellular particles (EXTRA) was also estimated. Data are expressed as proportions (means ± deviations of results from triplicate experiments) of each virus layer. (F) Quantification of core penetration. The intracellular virus particles (*n* > 500 for each condition) detected within endocytic vesicles (ENDO) or as cytosolic cores (CYTOSOL) were quantified in triplicate samples for both sets of conditions. Data are expressed as proportions (means ± deviations of results from triplicate experiments) of each virus category relative to the total number of intracellular particles.

To further investigate this finding, we performed EM analyses of sections of Vero cells infected by defective vE199Li^−^ or control vE199Li^+^ particles for 3 h. As shown in Fig. 9A and B, incoming particles lacking pE199L protein underwent normal endocytosis and particle disassembly, which involves the loss of the outer membrane and the protein capsid at multivesicular late endosomes (23). Importantly, most of the defective vE199Li^−^ particles were found to accumulate as “enveloped cores” (i.e., surrounded by the inner membrane) in lysosome-like structures (Fig. 9C) whereas naked cores were only minimally detected within the cytosol. In contrast, control infections performed with vE199Li^+^ particles led to the accumulation of significant amounts of cytosolic naked cores, which usually appeared grouped in clusters (Fig. 9D). These results are clearly consistent with a fusion defect of vE199Li^−^ particles. However, since fusion events of incoming ASFV particles are difficult to visualize by EM (likely because they occur rapidly and do not involve a “prominent” intermediate structure), we measured the structural changes that precede and follow viral fusion. Thus, we quantified the structural integrity of the endocytosed particles containing or lacking the pE199L protein (Fig. 9E) as well as the ability of both viruses to deliver naked cores to the cytosol (Fig. 9F). As shown in Fig. 9E, the disruption in the endosomes of the defective vE199Li^−^ particles, as judged by the loss of the outer membrane and the outer capsid, was similar to that found in control pE199L^+^ particles. In contrast, core penetration of defective vE199Li^−^ particles was reduced by a factor of 11.4 ± 2.7 compared to the control vE199Li^+^ particles (Fig. 9F). Taken together, these results indicate that pE199L protein is necessary for core delivery but not for the disassembly process that precedes the fusion of the inner viral membrane.

### Proteins pE199L and pE248R play nonredundant roles in core penetration.

**FIG 10:**
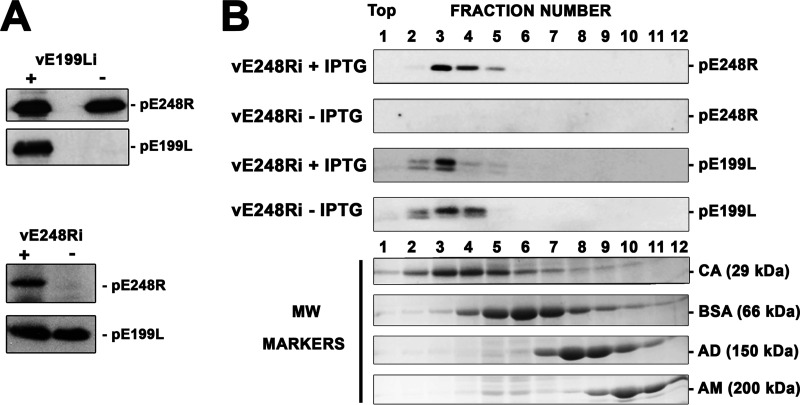
Nonredundant roles of proteins pE199L and pE248R in virus entry. (A) Equal amounts of control vE199Li^+^ and defective vE199Li^−^ virions (top) or vE248Ri^+^ and vE248Ri^−^ particles (bottom) were analyzed by immunoblotting with antibodies to pE199L and pE248R proteins. (B) Membrane fractions from Vero cells infected with recombinant vE248Ri in the presence (+IPTG) or absence (−IPTG) of inducer were subjected to detergent solubilization and analyzed by sucrose density gradient (5% to 20%) centrifugation. The fractions of both gradients were analyzed by electrophoresis and immunoblotting with antibodies to pE199L and pE248R proteins. As a reference, the sedimentation profiles of the molecular weight markers carbonic anhydrase (CA, 29 kDa), bovine serum albumin (BSA, 66 kDa), alcohol dehydrogenase (AD, 150 kDa), and β-amylase (AM, 200 kDa) are shown.

The recombinant vE199Li phenotype described above closely resembles that observed previously for a conditional lethal mutant (vE248Ri) of the pE248R inner envelope protein (23, 33). This led us to explore whether the absence of pE199L expression precludes the incorporation of pE248R into the viral particle, which would explain the common phenotype observed for the two recombinants. With this aim, purified vE199Li virus particles produced under permissive and nonpermissive conditions were analyzed by Western blotting for the presence of pE248R protein. As shown in Fig. 10A, the recombinant vE199Li^−^ lacking pE199L protein contained normal levels of pE248R. Reciprocally, defective recombinant vE248Ri^−^ lacking pE248R protein contained normal levels of pE199L. This indicates that each of these proteins can be independently recruited to the virus particle, where they play nonredundant roles in virus entry.

Finally, we explored the possibility that proteins pE199L and pE248R interact with each other, or through other proteins, to form higher-order complexes. With this aim, Vero cells infected for 24 h with recombinant vE248Ri in the presence or absence of IPTG were lysed and the derived membranes were extracted under mild conditions (1% nonionic, nondenaturing detergent IGEPAL CA-630) similar to those used for analyzing the VACV EFC. Then, the solubilized proteins were subjected to 5% to 20% sucrose density gradient sedimentation and the resulting gradient fractions were analyzed by immunoblotting performed with antibodies to pE199L and pE248R proteins. As shown in Fig. 10B, the sedimentation profiles of protein pE199L looked similar in both the absence and the presence of protein pE248R. Moreover, the sedimentation peaks of proteins pE199L (∼20 kDa) and pE248R (∼28 kDa) were comparable to that of marker carbonic anhydrase (∼29 kDa), which is consistent with their migration as monomers. Collectively, these results suggest that pE199L and pE248R proteins do not form a higher-order complex. However, the possibility cannot be excluded that such hypothetical complex may not be extracted from the viral membranes or may not be stable enough to be detected under the experimental conditions used.

## DISCUSSION

In accordance with its structural complexity, ASFV employs an intricate internalization pathway to infect the host cell. After virus uptake, the incoming particles undergo a stepwise, low-pH-driven disassembly process that leads to the loss of their outer membrane and outer capsid at multivesicular late endosomes (23). As a consequence, the inner envelope becomes exposed and merges with the endosomal membrane to deliver genome-containing viral cores to the cytosol. We have previously shown that ASFV protein pE248R is involved in this fusion process (23). The present report identifies a second viral protein, pE199L, which plays a key role in membrane fusion and core penetration.

Using an inducible ASFV recombinant, we have shown that protein pE199L is essential for virus replication, although its knockdown apparently does not interfere with the production of extracellular virus particles. Nevertheless, viral particles lacking pE199L are about 100-fold less infectious than the parental viruses, although they bind efficiently to the cell surface, being endocytosed and subsequently disassembled like the parental virions. Importantly, pE199L defective viral particles do not achieve membrane fusion and core release into the cytosol, accumulating within lysosome-like structures. The same defective phenotype has been reported for a conditional lethal mutant of the inner envelope protein pE248R (23, 33). Remarkably, the defective viruses lacking pE199L protein contain normal levels of pE248R and vice versa, which emphasizes the pivotal, nonredundant role of both proteins in membrane fusion.

As mentioned in the introduction, proteins pE199L and pE248R, which are highly conserved in all ASFV strains, display weak sequence similarity to some of the 11 transmembrane polypeptides that compose the membrane fusion machinery of poxviruses. Thus, protein pE199L resembles VACV EFC proteins A16, G9, and J5, whereas pE248R is similar to L1 and F9 (5). Notably, all 11 of the VACV EFC components accomplish essential, nonredundant roles, as deduced from the defective phenotype of their corresponding conditional lethal mutants. Similarly to the ASFV pE199L and pE248R proteins, the poxviral EFC proteins are not required for virus morphogenesis or virus binding but are essential for the penetration of the viral cores in the cytoplasm (26, 28). Like the poxviral EFC components, ASFV proteins pE199L and pE248R are located at the inner envelope that encloses the genome-containing core and behave as integral membrane proteins. Consistent with this, the protein sequences of pE248R and pE199L, and of their corresponding VACV orthologues, include a hydrophobic domain close to their C-terminal regions (ASFV protein pE248R is also N-myristoylated, like VACV proteins L1, A16, and G9). Furthermore, both groups of viral orthologues contain multiple conserved cysteines at their large N-terminal domains which form cytosolic intramolecular disulfide bonds. In the case of the poxviral EFC proteins, this unconventional formation of cytosolic disulfide bonds is catalyzed by a redox system formed by three virus-encoded thiol oxidoreductases, E10, A2.5, and G4, which are essential for virus replication (36). Interestingly, ASFV encodes a flavin adenine dinucleotide (FAD)-linked sulfhydryl oxidase, pB119L, that catalyzes the *in vitro* formation of disulfide bonds on different substrates (46). During ASFV infection, the sulfhydryl oxidase binds to protein pA151R, a viral polypeptide with a CXXC redox motif that, in turn, interacts with protein pE248R (46). It is also worth mentioning that deletion of the B119L gene greatly impairs *in vitro* replication of ASFV in swine macrophages and significantly affects virulence *in vivo* (47). Although the effect of B119L deletion on disulfide formation has not been addressed, it seems plausible that ASFV encodes a redox system, comprising pB119L and pA151R proteins, that accounts for disulfide bonding in the relatively reducing host cytoplasm, where ASFV replication takes place (46). In this context, the pE248R and pE199L inner envelope proteins seem probable final substrates of this redox machinery.

Taken together, the structural, biochemical, and functional analogies found among the pE199L and pE248R ASFV proteins and the VACV EFC components indicate that ASFV encodes a form of fusion machinery that is somehow reminiscent of that described for poxviruses. Interestingly, no other putative orthologue of VACV L1/F9 (apart from pE248R) and A16/G9/J5 proteins (apart from pE199L) has been detected either among the 15 transmembrane proteins found in the ASFV particle (11) or in the complete annotated viral genome. Also, we have not found any ASFV orthologue of the remaining VACV EFC proteins (A21, A28, G3, H2, L5, and O3), which, unlike L1/F9 and A16/G9/J5, contain a transmembrane domain at the N terminus. Hence, although the possibility of the presence of other viral components cannot be excluded, it is possible that the ASFV fusion apparatus represents a simplified version of that of poxviruses. In this regard, it is worth noting that other NCLDVs, namely, mimiviruses, iridoviruses, and ascoviruses, as well as the asfarvirus-like pithoviruses, kaumoebaviruses, and pacmanviruses, encode orthologues of VACV proteins L1/F9 and A16/G9/J5 but apparently do not encode orthologues of any of the other poxviral EFC components (5, 29). NCLDVs constitute a heterogeneous clade of large dsDNA viruses that derive from a common ancestor (6). Despite their structural differences, most NCLDVs contain a lipoprotein membrane that encloses the viral core and undergo membrane fusion for genome delivery during virus entry (23, 27, 28, 48). Strikingly, the two clusters of L1/F9 orthologs and A16/G9/J5 orthologs are the only known conserved NCLDV genes encoding transmembrane structural proteins (6, 35). Thus, it is tempting to speculate that all enveloped NCLDVs use similar strategies for membrane fusion, relying on this class of cysteine-rich transmembrane proteins. If so, this fusion machinery would represent one of the hallmarks of the ancestral NCLDV virion.

In contrast to the EFC found in VACV, we did not detect the presence of proteins pE199L and pE248R in higher-order structures from detergent-solubilized proteins of infected cell membranes. This could possibly be explained by the difficulty encountered in efforts to extract the hypothetical ASFV EFC under the assayed conditions or by its intrinsic instability. Also, the possibility of a very transient association of the putative EFC components (i.e., during the membrane fusion event only) cannot be excluded. In regard to the VACV EFC, it is thought that this complex is held together by multiple interactions among 9 of the 11 components and that the remaining L1 and F9 proteins (the orthologues of pE248R) are peripherally or weakly associated with this “core” structure (26, 28, 49). Indeed, neither F9 nor L1, each of which is a relatively abundant protein in VACV-infected cells and virions, was detected by mass spectroscopy analysis of the immunoaffinity purified EFC but was detected only by sensitive Western blotting. Also, there is no evidence of a direct interaction between the VACV orthologues of pE248R (L1/F9) and those of pE199L (A16/G9/J5). Collectively, these data might explain the lack of detection of a putative complex containing pE199L and pE248R in ASFV.

Very little is known about the molecular mechanisms underlying membrane fusion in enveloped NCLDVs. In general, viral membrane fusion is mediated by specialized viral membrane-anchored proteins containing a short stretch of hydrophobic amino acids which is able to induce the lipid destabilization required for membrane merging. On the basis of their primary sequence, none of the EFC components of VACV or ASFV resemble known fusion proteins of other enveloped viruses and the identity of the actual fusogenic peptide remains elusive. At present, only the three-dimensional (3D) structure of VACV L1 and F9 proteins has been elucidated. X-ray crystallography indicates that the two EFC paralogues display similar overall patterns of folding consisting of a bundle of α-helices packed against two pairs of β-sheets (31, 32). This particular pattern of folding, which is stabilized by disulfide bonds that covalently link the helices and sheets, is not found in other viral or cellular proteins. Additional structural studies, possibly investigating the EFCs or derived subcomplexes, are needed to understand the molecular mechanisms of membrane fusion in enveloped NCLDVs. In this regard, recent work using cryo-EM methods has begun to elucidate the molecular architecture of the ASFV virion with near-atomic resolution (7–9). Importantly, the VACV EFC has been shown to adopt a polarized distribution within the viral particle that is crucial for its activity (50). Whether the same concept applies to ASFV, with a radically different, icosahedral particle structure and possibly a reduced form of fusion machinery, is an impending issue to be resolved by structural approaches in the near future.

ASFV is presently causing a global animal health emergency because of its devastating effects on the pig populations in South Asia and Western Europe (3). The lack of vaccines or effective therapies against ASFV urges the need to address the existing relevant gaps in knowledge of the basic biology of its infection cycle. In this context, characterization of the viral components of the fusion machinery brings relevant information on ASFV entry and provides novel viral targets for the development of vaccines or antiviral strategies that block the first stages of ASFV replication.

## MATERIALS AND METHODS

### Cells and viruses.

Vero cells from African green monkey kidney tissue were obtained from the American Type Culture Collection and grown in Dulbecco’s modified Eagle’s medium (DMEM) containing 10% fetal bovine serum (FBS), which was reduced to a 2% concentration during viral infection. Swine alveolar macrophages, which derived from our own stock stored in liquid nitrogen, were seeded in DMEM supplemented with 10% heat-inactivated swine serum, 2 mM l-glutamine, 50 mg/ml gentamicin, and nonessential amino acids. All cell types were cultured at 37°C in a 5% CO_2_ atmosphere.

Vero cell-adapted ASFV strain BA71V and recombinant, BA71V-derived virus vE248Ri, which contains an inducible copy of the E248R virus gene, have been described before (33). All ASFV stocks used for virus entry assays were purified by the Percoll method (51), and their purity and integrity were checked by gel electrophoresis analysis as well as by negative-staining EM. For nonentry studies, infection supernatants clarified by low-speed centrifugation (1,000 × *g* for 10 min) were used as virus inocula, unless otherwise specified.

### Antibodies.

Rabbit polyclonal antibodies against ASFV polypeptides p150, p37, and p34 (derived from polyprotein pp220 [CP2475L]) have been described previously, as have those recognizing p15 and p35 (derived from polyprotein pp62 [CP530R]); p72 (B646L); p49 (B438L); p17 (D117L); p12 (O61R); pE248R, pEP402R/CD2v, p32 (CP204L), and pA104R (11, 33, 40, 42, 52, 53); and the rat sera to protein pE183L (42); as well the mouse monoclonal antibodies (MAb) against proteins p150 (clone 17A.H2), p72 (19B.A2), p12 (24BB7), and p24 (17E.H10) (54). The mouse MAb antibodies for α-tubulin (B-5-1-2) and β-actin (AC-15) were purchased from Sigma, the antibody for GM130 (35/GM130) was from BD Transduction Laboratories, and the antibody for CD63 (H5C6) was from the Developmental Studies Hybridoma Bank (University of Iowa). The rabbit IgG anti-bovine serum albumin (anti-BSA) (A11133) was purchased from Molecular Probes, and rabbit antibody to AKT was purchased from Abcam.

For the preparation of an antibody against the viral protein pE199L, the corresponding ORF was cloned in plasmid pRSETA and expressed in Escherichia coli. Inclusion bodies containing recombinant pE199L were dissociated and electrophoresed on 12% polyacrylamide gels. Then, the pE199L band was excised and used as an immunogen to raise polyclonal antibodies in rats.

### Generation of an ASFV recombinant virus for inducible expression of *E199L* gene.

A scheme of the ASFV recombinant vE199Li in which the expression of the *E199L* gene is under the control of the E. coli *lac* operator/repressor system is shown in Fig. 2A. In this virus, *E199L* expression is driven by the synthetic promoter p72I*, a highly repressible inducible promoter in which operator sequence O1 is separated by 2 bp from the strong late viral promoter p72.4 (2). To obtain this virus, plasmid pE199L was constructed by Gibson assembly (55) in sequential steps. In the final construct, the following elements are included: (i) a left-flanking region of 2,080 bp containing the complete E199L ORF and upstream sequences (nucleotides 147582 to 149581 of the BA71V genome); (ii) an inducible cassette containing the inducible promoter, a copy of the E. coli *lacI* repressor under the control of an early/late ASFV promoter (pU104L), and the complete coding sequence of the reporter β-glucuronidase gene under the control of a strong late ASFV promoter (p72.4); and (iii) a right-flanking region of a 2,035-bp DNA fragment (nucleotides 149404 to 151438 of the BA71V genome) containing the complete E165R ORF and downstream sequences. The latter fragment also includes the 172-bp region immediately upstream of E165R that encompasses its promoter with the first transcription initiation position for E165R (56), as well as the complete E199L promoter and the first 150 bp of amino acids of its coding sequence. To avoid the expression of a fusion protein corresponding to this small E199L-derived fragment, the two in-frame starting codons that were present in the original sequence were removed by point mutation. The absence of unwanted mutations in the final construct was confirmed by conventional Sanger sequencing. A detailed description of the cloning steps and the final sequence of the generated plasmid are available upon request.

Recombinant virus vE199Li was generated essentially as described previously (38) with minor modifications. Briefly, Vero cells were transfected with linearized plasmid pE199Li and infected with BA71V virus in the presence of different concentrations of isopropyl-β-d-thiogalactopyranoside (IPTG). At 72 h postinfection (hpi), the cells were harvested and recombinant virus vE199Li was isolated by sequential rounds of plaque purification in the presence of IPTG at 1 mM, a concentration that was found to be optimal for the production of the recombinant virus. To confirm the genetic structure of the vE199Li recombinant, viral DNA was purified and subjected to next-generation sequencing (NGS). Briefly, extracellular virus was semipurified, concentrated by ultracentrifugation through a 36% sucrose cushion, and digested using nonencapsidated DNA with a mixture of micrococcal nuclease S7 and DNase I. The sample was then treated with proteinase K, and viral DNA was finally obtained by phenol extraction and ethanol precipitation in the presence of carrier glycogen. Libraries were prepared using 250 ng of purified viral DNA and a Nextera DNA Flex library prep kit (Illumina), following the manufacturer’s instructions. Fragments of 500 to 600 bp were obtained after electrophoresis of the library in a 1.5% gel and DNA extraction and purification performed with a MinElute gel extraction kit (Qiagen). NGS was performed on a MiSeq sequencer (Illumina) in a 2 × 300 run with MiSeq reagent kit v3 (Illumina) (600 cycles). Reads obtained were mapped to the expected vE199Li reference genome with Bowtie2 aligner software using default parameters. The average coverage obtained for a covered base was 221×, and the corresponding sequence analysis was used to discard any additional and nondesirable mutations in vE199Li genome.

### Plaque assay.

Vero cell monolayers, seeded in six-well plates, were infected with recombinant vE199Li or with parental BA71V as a control. After 1 h, the inoculum was removed and the cells were overlaid with DMEM containing 0.6% Noble agar and 2% FBS in the presence or absence of 1 mM IPTG. The medium was removed 5 to 7 days later after formaldehyde fixation, and the monolayers were stained with 1% crystal violet.

### One-step virus growth curves.

Vero cell monolayers, seeded in 24-well plates, were infected with 5 PFU/cell of recombinant vE199Li or parental BA71V. After a 1-h adsorption step, the cells were incubated in medium supplemented with 2% FBS in the presence or absence of 1 mM IPTG. Finally, the infected cells were harvested together with their culture supernatants at different times postinfection, sonicated, and titrated by plaque assay in the presence of 1 mM IPTG.

### Analysis of membrane association of protein pE199L.

Mock- and ASFV-infected cells were fractionated at 20 hpi into nuclear, cytoplasmic, and membrane fractions, according to the instructions provided with a Minute plasma membrane protein isolation and cell fractionation kit (Invent Biotechnologies). For alkaline carbonate extraction, membrane fractions were treated with 0.1 M Na_2_CO_3_ (pH 11.5), incubated for 30 min at 4°C, and subsequently centrifuged for 10 min at 16,000 × *g*. The supernatant and the sediment were dissociated by the use of electrophoresis sample buffer. For treatment with Triton X-114, the membrane fraction was resuspended in a mixture containing 2% Triton X-114, 150 mM NaCl, and 10 mM Tris-HCl (pH 7.5), incubated for 10 min at 4°C, and then transferred to 30°C for 20 min. The samples were briefly centrifuged to separate the lower detergent-rich phase from the upper phase, and the two phases were dissociated as described above. Equivalent amounts of the resulting fractions were analyzed by Western immunoblotting with a rat anti-pE199L antibody.

### Analysis of disulfide bonds in protein pE199L.

ASFV-infected Vero cells were incubated on ice for 10 min with phosphate-buffered saline (PBS) in the presence or absence of a 20 mM concentration of the sulfhydryl modifying agent N-ethylmaleimide (NEM; Sigma-Aldrich). Then, the samples were dissociated with Laemmli buffer in the presence or absence of 100 mM dithiothreitol (DTT), analyzed by electrophoresis on 12% SDS-PAGE gels, and immunoblotted with an anti-pE199L antibody.

### Immunofluorescence microscopy.

Vero cells or swine macrophages, seeded on glass coverslips, were infected with purified parental ASFV virus or recombinant vE199Li for the indicated times, washed with PBS, and fixed with methanol at –20°C for 5 min or with 4% paraformaldehyde (PFA)–PBS for 15 min at room temperature (RT). In the latter case, infected cells were permeabilized with 0.1% saponin–PBS for 5 min at RT, treated with 50 mM NH_4_Cl for 5 min to quench free aldehydes, and blocked with 10% FBS for 5 min. The permeabilized infected cells were incubated for 45 min at RT with primary antibodies and for 30 min at RT with Alexa Fluor-labeled secondary antibodies (Thermo Fisher). Primary antibodies were diluted as follows: rat anti-E199L at 1:500, mouse MAb anti-p72 (19BA2) at 1:500, rabbit anti-p12 at 1:500, and mouse MAb anti-CD63 at 1:500. Secondary antibodies were diluted 1:500. All antibody dilutions were prepared in 5% FBS–PBS. Cell nuclei were labeled with Hoechst 33258 (5 μg/ml). Coverslips were mounted with ProLong glass antifade mountant (Thermo Fisher) on microscope slides. Images were recorded with a Leica DMI6000B automated inverted microscope equipped with a Hamamatsu Orca R2 digital camera or a Zeiss LSM710 multiphoton laser microscope using a 100× Plan-Apochromat lens objective (numerical aperture [NA], 1.4). Quantitative colocalization analysis was performed with the BIOP JACoP plugin (https://c4science.ch/w/bioimaging_and_optics_platform_biop/image-processing/imagej_tools/jacop_b/) of Fiji software by calculating the Manders split colocalization coefficients (M1 and M2) after applying automated Otsu thresholding. These values, which range from 0 for no colocalization to 1 for perfect colocalization, express the fraction of intensity corresponding to one color channel that overlaps the signal from the other channel. At least 10 optical sections corresponding to different z-stacks of cell profiles were analyzed per condition. M1 and M2 coefficients are expressed as mean values ± standard deviations (SD).

### Virus egress assay.

Vero cells were infected with recombinant vE199Li (5 PFU/cell) in DMEM containing 0.2% FBS in the presence or absence of 1 mM IPTG. At the indicated times, the extracellular medium was collected and centrifuged at low speed (1,000 × *g* for 5 min) to remove any cell debris. Then, the extracellular proteins were precipitated with 10% cold trichloroacetic acid (TCA) and sedimented at 15,000 × *g* for 20 min at 4°C. Finally, protein extracts were dissociated in electrophoresis sample buffer and analyzed by SDS-PAGE followed by Western immunoblotting performed with antibodies against viral structural proteins pE199L, pEP402R/CD2v, and p150. The serum albumin levels for each lane, which were estimated with an anti-BSA antibody, were used as protein loading controls. The expression levels of each protein were estimated by densitometry and normalized by the BSA content.

### Early and late infection analysis of purified vE199Li^−^ particles.

Vero cells or swine macrophages seeded on 24-well plates were incubated with 1 PFU/cell of Percoll-purified extracellular parental BA71V and with equivalent amounts of extracellular vE199Li particles produced in the presence or absence of 1 mM IPTG. After 1 h of adsorption at 37°C, the infections were carried out in the presence of IPTG for the indicated times and examined by phase-contrast microscopy for the assessment of cytopathic effect or dissociation was performed with sample buffer for the assessment of the early (p32) and late (p72) viral protein expression levels by Western immunoblotting.

### Virus binding and internalization assays.

For cell binding assays, Vero cells seeded on glass coverslips were incubated for 2 h at 4°C with equivalent amounts of purified control vE199Li^+^ or defective vE199Li^−^ particles. After extensive washes with cold PBS, the cells were fixed for 3 min with methanol at −20°C and analyzed by immunofluorescence with a rabbit antibody against the p72 viral protein and a mouse MAb against the p24 (17E.H10) plasma membrane marker followed by Alexa-labeled secondary antibodies. Nuclei were labeled with Hoechst 33258 (blue). The number of bound virus particles per cell for each condition (which was typically in the range of 50 to 150) was quantified using maximum intensity projections of 20 cell areas (performed in triplicate) acquired with an oil immersion 63× objective. Data were expressed as the percentage of virus adsorption relative to control vE199Li^+^ infections (means of results from three independent experiments ± SD).

To quantify virus uptake, Vero cells seeded on 48-well plates were incubated with equal amounts of control vE199Li^+^ (10 PFU/cell) or defective vE199Li^−^ virus particles during 2 h at 37°C in the presence or absence of 50 mM Dynasore (Dyn). Then, the cells were incubated with trypsin-EDTA for 5 min at 37°C to remove noninternalized particles and fixed with 4% PFA for 15 min at RT. After permeabilization with 0.1% TX-100 (5 min at RT), the internalized particles were immunolabeled with rabbit anti-p12 antibody followed by an Alexa Fluor 488-conjugated anti-rabbit antibody. Finally, the cells were resuspended in fluorescence-activated cell sorter (FACS) buffer (PBS, 0.01% sodium azide, 0.5% BSA) and the fluorescence was quantified by flow cytometry using a FACSCalibur flow cytometer (BD Sciences). All FACS analyses were performed in triplicate, and the results are displayed as the mean fluorescence intensity normalized to the control infection intensity in the absence of Dynasore and trypsin treatments.

### Density gradient centrifugation.

Vero cells infected for 18 h with recombinant vE248Ri in the presence or absence of IPTG were fractionated into nuclear, cytoplasmic, and membrane fractions as described above. Membrane fractions were resuspended in 1% nonionic, nondenaturing detergent Igepal Co-630 (Sigma)–PBS containing a protease inhibitor cocktail (Sigma) and incubated for 60 min on ice with occasional vortex mixing. Detergent-solubilized proteins were centrifuged in a 5%-to-20% (wt/vol) sucrose density gradient (containing 0.1% Igepal Co-630–PBS) for 12 h at 40,000 rpm and 5°C using a swinging-bucket TLS-55 rotor (Beckman). After gradient fractionation, the proteins were precipitated with 10% trichloroacetic acid under cold conditions, washed with acetone, and dissociated with Laemmli buffer. Finally, the proteins were electrophoresed on 12% SDS-PAGE gels and immunoblotted with anti-pE199L and anti-pEE248R antibodies.

As a reference, the following molecular weight standards (Sigma) were also analyzed by sucrose gradient centrifugation in separated tubes: carbonic anhydrase (29 kDa), bovine serum albumin (66 kDa), alcohol dehydrogenase (150 kDa), and β-amylase (200 kDa). Protein markers were electrophoresed as described above and stained with Coomassie blue.

### Western blotting.

The cell and virus samples were dissociated in Laemmli buffer (2% SDS, 100 mM DTT, 125 mM Tris-HCl, pH 6.8), heated at 90°C for 5 min, and electrophoresed on 12% SDS-polyacrylamide gels. The proteins were transferred to polyvinylidene difluoride (PVDF) membranes (Bio-Rad) (0.2-μm pore size). Membranes were incubated overnight at 4°C with the primary antibodies and for 1 h at RT with the corresponding secondary antibodies conjugated to horseradish peroxidase (GE Healthcare). Blots were developed with enhanced chemiluminescence Prime Western blotting detection reagent (GE Healthcare), imaged with an ImageQuant LAS 4000 mini imager (GE Healthcare), and quantified with the ImageQuant TL software package (GE Healthcare).

### Transmission electron microscopy.

For virus assembly studies, Vero cells or swine macrophages seeded on 60-mm-diameter plates were infected with 5 PFU/cell of ASFV (BA71V strain) in DMEM containing 2% FBS or 10% swine serum, respectively. At the indicated times, cells were fixed with 4% PFA–2% glutaraldehyde (GLA)–0.1 M phosphate buffer (PB, pH 7.4) for 90 min at RT. Postfixation was carried out with 1% OsO_4_–0.8% K_3_Fe(CN)_6_–water at 4°C for 1 h. Samples were dehydrated with acetone and embedded in epoxy (TAAB 812 resin; TAAB Laboratories) according to standard procedures. After polymerization, 80-nm-thick (ultrathin) sections were obtained and stained with uranyl acetate and lead citrate according to standard procedures. Samples were examined in a JEOL JEM-1010 electron microscope operating at 80 kV. Images were recorded with a TemCam-F416 digital camera (4,000 pixels by 4,000 pixels) from TVIPS.

For virus entry studies, Vero cells seeded on 48-well plates were infected by spinoculation during 30 min at 650 × *g* with 100 to 200 PFU/cell of recombinant vE199Li in DMEM containing 2% FBS. At the indicated times, the cells were fixed with 4% PFA and 2% GLA and postfixed with 1% OsO_4_ and 0.8% K_3_Fe(CN)_6_ as described above. Samples were dehydrated with ethanol and subjected to *in situ* flat embedding in epoxy resin as described above. After polymerization, resin sheets containing the cell monolayers were detached from the substrate and mounted onto resin blocks to obtain orthogonal 80-nm-thick (ultrathin) sections. The sections were deposited onto slot grids and stained as described above. For quantification of ASFV-containing vesicles and cytosolic virus cores, ultrathin orthogonal sections were systematically screened at magnifications of ×3,000 to ×5,000 along a linear track from one end of the EM slot grid to the other. About 50 to 100 cell profiles containing more than 500 intracellular viral particles were analyzed per condition in triplicate samples. For quantification of virus disassembly, more than 100 endocytosed particles per sample were analyzed at magnifications of ×20,000 to ×40,000 for the presence of the inner membrane, outer capsid, and outer membrane (23). Extracellular cell-associated virus particles (*n* > 50 per sample and condition) were also quantified as a reference. Data were expressed as the frequency (means ± deviations of results from triplicate experiments) of each virus layer in the endocytosed particles per condition.

For immunoelectron microscopy, infected cells were fixed *in situ* with 4% PFA–0.1 M PB for 2 h at RT and kept in 1% (wt/vol) PFA in PB at 4°C. Subsequently, cells were embedded in 10% (wt/vol) gelatin and subjected to cryoprotection in sucrose (2.3 M) overnight. Specimens were rapidly frozen in liquid nitrogen and cryosectioned with a Leica EM FCS cryoultramicrotome at −120°C. For immunogold labeling, thawed 90-nm-thick cryosections were incubated with 20 mM glycine for 5 min at RT to quench free aldehyde groups and with 10% FBS for 5 min at RT to block nonspecific binding. Then, sections were incubated with a rat anti-pE199L antibody for 30 min at RT followed by a goat anti-rat secondary antibody conjugated to 10-nm-diameter gold particles (British Biocell, United Kingdom) for 30 min at RT. The primary antibodies and gold conjugates were diluted in PBS containing 5% FBS. Sections were stained with a mix of 1.8% methylcellulose and 0.4% uranyl acetate before visualization. For quantification of pE199L labeling, the radial distribution of the gold particles was calculated for intracellular virus profiles (*n* = 50), displaying well-defined hexagonal outlines.

### Sequence analysis.

Possible orthologues of all the proteins belonging to the Cluster of orthologous genes (COG) “virion-associated membrane protein” identified previously by Iyer et al. (5) were searched among NCLDVs using standard blastp searches of the Uniprot portal (https://www.uniprot.org). A representative list of identified proteins (limited to a single orthologue per virus family) was used for sequence alignment, which was performed using the Clustal omega tool at the EMBL European Bioinformatics Institute site (https://www.ebi.ac.uk/). The aligned proteins (including the corresponding virus of origin, annotated names, and Uniprot accession numbers) were as follows: VACV_A16 (vaccinia virus strain Western Reserve [WR], virion membrane protein A16, YP_233018.1); VACV_G9 (vaccinia virus strain WR, myristoylated protein G9, P07611); VACV_J5 (vaccinia virus strain WR, protein J5, P07618); IIV-6_337L (invertebrate iridescent virus 6, putative membrane protein 337L, Q91FI7); SfAV-1a_054 (Spodoptera frugiperda ascovirus 1a, 30.3-kDa myristylated membrane protein-like, Q0E547); PithoV_02210 (pithovirus LCPAC101, entry-fusion-complex G9/A16, A0A481Z4E7); KaumV_00037 (kaumoebavirus, uncharacterized protein, A0A1V0CNF2); KNV_5_65 (klosneuvirus KNV1, myristylated protein, A0A1V0SKX5); PACV_2 (pacmanvirus A23, uncharacterized protein, A0A1X6WEJ1); and ASFV_E199L (African swine fever virus strain Badajoz 1971 Vero-adapted, cysteine-rich protein E199L, Q65198).

### Statistical analysis.

Unless otherwise indicated, the data are representative of results from at least three independent experiments, and values are given as the means of triplicates ± standard deviations (SD).

### Data availability.

Files containing sequencing reads were deposited at ENA (European Nucleotide Archive) under project number PRJEB36979.
